# Type 2 diabetes and cardiometabolic risk may be associated with increase in DNA methylation of *FKBP5*

**DOI:** 10.1186/s13148-018-0513-0

**Published:** 2018-06-19

**Authors:** Robin Ortiz, Joshua J. Joseph, Richard Lee, Gary S. Wand, Sherita Hill Golden

**Affiliations:** 1 Departments of Internal Medicine & Pediatrics, Johns Hopkins University School of Medicine, 600 North Wolfe Street, Harvey Bldg. Rm 805, Baltimore, MD 21287 USA; 2 Division of Endocrinology, Diabetes, and Metabolism, Department of Medicine, The Ohio State University Wexner Medical Center, 566 McCampbell Hall, 1581 Dodd Drive, Columbus, OH 43210 USA; 3 Department of Psychiatry and Behavioral Sciences, Johns Hopkins University School of Medicine, 720 Rutland Ave. Ross Bldg. 1068, Baltimore, MD 21205 USA; 4 Division of Endocrinology, Diabetes and Metabolism, Department of Medicine, Johns Hopkins University School of Medicine, 1830 E. Monument Street Suite 333, Baltimore, MD 21287 USA

**Keywords:** *FKBP5*, Methylation, Epigenetics, Cortisol, Diabetes, Cardiovascular disease, Obesity, Body mass index (BMI), Waist circumference, Hemoglobin A1c

## Abstract

**Background:**

Subclinical hypercortisolism and hypothalamic-pituitary-adrenal (HPA) axis dysfunction are associated with type 2 diabetes (T2DM), cardiovascular disease, and metabolic dysfunction. Intronic methylation of *FKBP5* has been implicated as a potential indicator of chronic cortisol exposure. Our overall objective in this study was to determine the association of chronic cortisol exposure, measured via percent methylation of *FKBP5* at intron 2, with percent glycosylated hemoglobin (HbA1c), low-density lipoprotein cholesterol (LDL-cholesterol), waist circumference (WC), and body mass index (BMI), in a clinic-based sample of 43 individuals with T2DM.

**Results:**

Greater percent methylation of the *FKBP5* intron 2 at one CpG-dinucleotide region was significantly associated with higher HbA1c (*β* = 0.535, *p* = 0.003) and LDL cholesterol (*β* = 0.344, *p* = 0.037) and a second CpG-dinucleotide region was significantly associated with higher BMI and WC (*β* = 0.516, *p* = 0.001; *β* = 0.403, *p* = 0.006, respectively).

**Conclusions:**

*FKBP5* methylation may be a marker of higher metabolic risk in T2DM, possibly secondary to higher exposure to cortisol. Further work should aim to assess the longitudinal association of *FKBP5* with cardiovascular disease and glycemic outcomes in T2DM as a first step in understanding potential preventive and treatment-related interventions targeting the HPA axis.

**Electronic supplementary material:**

The online version of this article (10.1186/s13148-018-0513-0) contains supplementary material, which is available to authorized users.

## Background

Subclinical hypercortisolism and hypothalamic-pituitary-adrenal (HPA) axis dysfunction are associated with type 2 diabetes (T2DM), cardiovascular disease, and metabolic dysfunction [1–3]. While recent research has shown alterations of the cortisol diurnal profile in those with T2DM, further research in this area has been hindered by lack of a chronic total cortisol (glucocorticoid) exposure measure. Recently, epigenetic modification of HPA axis-targeted genes has been identified as a potential measure of chronic exposure to cortisol and HPA axis dysfunction [4, 5]. Understanding the role of HPA dysfunction, assessed via epigenetic modifications, in metabolic disorders, T2DM, and cardiovascular disease, may lead to a unifying pathophysiology, thus setting the stage for preventive and therapeutic interventions targeting the HPA axis. It has been proposed that identifying a target, such as a specific modulator or receptor, could allow for restoration of HPA axis function to a homeostatic physiological state thereby reversing subclinical hypercortisolism and its associated comorbidities including metabolic syndrome, T2DM, and cardiovascular disease [6].

The FK506-binding protein 51 kDa (*FKBP5*) gene is a modulator, or co-chaperone, of the glucocorticoid receptor by limiting translocation of the receptor complex to the nucleus [7]. An observation that changes in *FKBP5* methylation at intron 2, specifically, were associated with shaping the human brain and function was one of the first demonstrations of the epigenetic interaction between genetics and environment [8]. *FKBP5* has been linked to metabolic dysfunction including regulation of body weight after bariatric surgery and insulin resistance in non-diabetic individuals [9, 10]. Epigenetic modification via loss of intronic methylation of *FKBP5* has been associated with Cushing’s syndrome and can serve as an indicator of chronic exposure to cortisol [11]. Cushing’s can often manifest with cardiovascular and metabolic dysfunction including truncal obesity, hypertension, and glucose intolerance. *FKBP5* methylation has also been associated with neuropsychiatric disease [8, 12]. However, *FKBP5* methylation has not been studied in individuals with T2DM, or in the context of specific measures of metabolic and cardiovascular disease. Given its importance in the function of the HPA axis via regulation of the glucocorticoid receptor, if *FKBP5* is shown to be associated with metabolic syndrome, T2DM, and cardiovascular disease, it may serve as a promising target for interventions aimed at restoring HPA axis function.

Our overall objective in this study was to determine the association of chronic cortisol exposure, measured via percent methylation of *FKBP5* at, specifically, intron 2, as was studied by Klengel, et al. [8], with markers of metabolic and cardiovascular disease in those with T2DM. Specifically, the association of percent methylation of *FKBP5* with percent glycosylated hemoglobin (HbA1c), low-density lipoprotein cholesterol (LDL cholesterol), body mass index (BMI), and waist circumference (WC), which is a surrogate for central obesity and an independent risk factor for cardiovascular disease [13]. We further explored the association between methylation of *FKBP5* and (1) cardiovascular disease risk modifier, exercise, and (2) the presence of cardiovascular disease, assessed by the need for coronary artery disease intervention.

## Methods

### Aims

The aim of this study was to primarily assess methylation of *FKBP5* at intron 2 with markers of metabolic and cardiovascular disease in those with T2DM including HbA1c, LDL cholesterol, BMI, and WC. A secondary aim was to assess the potential relationship between *FKBP5* and exercise as well as a history of cardiovascular procedures in the study cohort.

### Study participants

MinD, a study assessing the crude prevalence of minor depressive disorder (MinD) in a clinic-based population of 128 consented adults with T2DM, participant data was used for this analysis. Because the primary aim of the MinD study focused on data assessing minor depression prevalence and comorbidity with associated cardiovascular and metabolic measures in subjects with T2DM, control subjects without diabetes were not included in the original design. Further, participants taking antipsychotic medication or glucocorticoids were excluded as both medications may be associated with major mood disorders. The detailed methods of study design were previously described elsewhere and are described here briefly [14]. Individuals with physician-confirmed T2DM who were 18 years of age or older were recruited between 2011 and 2013 from the Diabetes Center clinics at Johns Hopkins Hospital during routine visits. Patients who were willing to participate in the study provided written informed consent. This study was approved by the Johns Hopkins University School of Medicine Institutional Review Board.

### Exposures and outcomes samples

Baseline information was obtained during clinic visits using standardized questionnaires including demographics, race, level of education, and annual income. Calibrated devices were used by certified technicians and nurses to measure participants’ weight, WC (average of two measurements around the umbilicus), and height. BMI was calculated as weight (kilograms)/height^2^ (m^2^). Resting seated blood pressure (BP) was measured twice at 5-min intervals using an appropriately sized cuff with standard Hawksley random-zero instruments, and measurements were averaged for analysis. Hypertension was defined as systolic BP of 140 mmHg or greater, diastolic BP of 90 mmHg or greater, or use of antihypertensive therapy. HbA1c and lipid panels (including low-density lipoprotein, triglycerides, and high-density lipoprotein) were also collected and analyzed by Johns Hopkins Clinical Core Laboratory with LDL cholesterol calculated from total cholesterol.

A secondary aim of the MinD data collection was to assess the relationship of *FKBP5* with measures of cardiometabolic risk in diabetes. Therefore, a subset of patients consented to provide blood samples to be used for future research purposes. Only subjects who did not consent to blood collection of DNA for research purposes were excluded. DNA was extracted from the blood and stored at − 20 °C, until epigenetic analyses were conducted.

### Bisulfite pyrosequencing for DNA methylation (DNAm) quantification

Two sets of bisulfite polymerase chain reaction (PCR) primers were designed to target each of two regions in the second intron of the human *FKBP5* gene. The two regions flanked a glucocorticoid response element (GRE) [15] and contained consecutive cytosine-guanine dinucleotides (CpGs) implicated in previous studies [8, 11]. The coordinates of the nine CpGs on chromosome 6 are Chr6: 35,606,441–35,606,662 (CpG1-4) and Chr6: 35,609,542–35,609,666 (CpG5-9), according to the UCSC Genome Browser build GRCh37.hg19 assembly [16]. Two rounds of a nested PCR amplification were performed. In the first PCR, 3.5 μL bisulfate converted DNA was used. In the second PCR, 2 μL template from the PCR was used. One of the nested bisulfite primers was biotinylated and HPLC-purified, allowing it to bind to sepharose beads and become single-stranded, in preparation for bisulfite pyrosequencing. The single-stranded amplicons were annealed to pyrosequencing primers and subjected to primer extension and nucleotide incorporation using the PyroMark Q96 MD pyrosequencer (Qiagen). The pyrosequencer QCpG program determined percent DNA methylation (DNAm) at all of the CpG dinucleotides downstream of the annealed primer at > 90% precision.

### Statistical analysis

Subject demographics were analyzed using descriptive statistics. Outliers in methylation assays were identified if the value was greater than three standard deviations away from the mean suggesting sub- or supra-physiologic levels and likely erroneous. Multivariable linear regression was performed to examine the association of percent DNAm of *FKBP5* (dependent variable) with HbA1c, LDL cholesterol, BMI, and WC (independent variables). Age, sex, and race were included as covariates in all regression analyses, based on previous cortisol analyses [1, 17]. Beta regression coefficients with a *p* value < 0.05 were considered statistically significant. Given this was established as a hypothesis-generating study, no adjustments were made for multiple comparisons as such tests were considered too conservative for correlated hypotheses as we propose here. For all independent variables with significant beta regression coefficients, basic correlation R values were used to assess effect size using Cohen’s effect size such that *r* = 0.1 was considered of small effect size, *r* = 0.3 was considered of medium effect size, and *r* = 0.5 was considered of large effect size [18]. Based on preliminary results, a secondary analysis was completed including a multivariate regression with the number of cardiovascular procedures as a dependent variable and another with the number of days of physical activity greater than 30 min as an independent variable. All statistical analyses were performed using IBM SPSS Statistics Version 23.

## Results

A total of 65 subjects had given consent for blood samples for epigenetic analysis. After excluding individuals with missing data on HbA1c (*n* = 5), LDL cholesterol (*n* = 11), BMI (*n* = 1), and WC (*n* = 11), 43 participants were included in the analysis (Additional file 1: Figure S1). The participants were 48.8% female, 60.5% non-Hispanic black with a mean age of 61.9 years (SD ±9.0 years); other demographics are described in Table 1. Given that all participants had diabetes and were on anti-hypertensive medication, 81.4% of subjects with WC ≥ 94 cm in men or ≥ 80 cm in women, therefore, met the National Cholesterol Education Program Adult Treatment Panel III (NCEP:ATPIII) criteria for metabolic syndrome [19].

**Table 1:** Participant demographic characteristics (*n* = 43)

Characteristic	Percentage or mean (standard deviation)
Sex (%)	
Female	48.8% (*n* = 21)
Race (%)	
Non-Hispanic White	37.2% (*n* = 16)
Non-Hispanic Black	60.5% (*n* = 26)
Asian	2.3% (*n* = 1)
Age (years)	61.9 ± 9.0
WC (cm)	109.7 ± 21.6
WC ≥ 94 cm (men) or 80 cm (women) (%)	81.5% (*n* = 35)
BMI (kg/m^2^)	34.1 ± 8.3
Antihypertensive medication use (%)	100% (*n* = 43)
HbA1c (%)	8.3 ± 1.6
LDL cholesterol (mg/dL)	89 ± 40
Cardiovascular procedure (%)*	
Catheterization	11.6% (*n* = 5)
Percutaneous coronary intervention (PCI)	4.7% (*n* = 2)
Coronary artery bypass surgery (CABG)	7.0% (*n* = 3)
PCI and CABG	4.7% (*n* = 2)

*Collectively this represents the total number of individuals included in the FKBP5 analysis model with a history of cardiovascular procedures (*n* = 12)

**Fig. 1:**
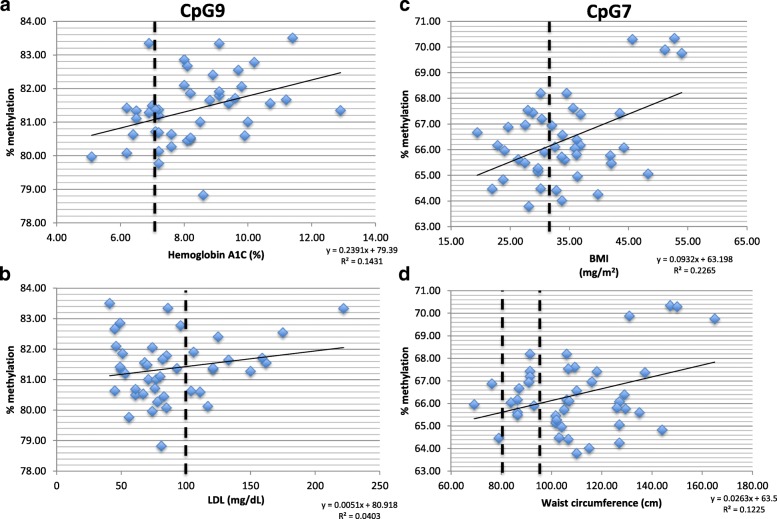
*FKBP5* methylation associated with cardiometabolic risk in individuals with diabetes. **a** Greater percent DNA methylation of the *FKBP5* intron 2 CpG9-dinucleotide (CpG9) (coordinates chr6: 35,609,542) was significantly associated with higher HbA1c (*β* = 0.535, *p* = 0.003), with a medium to large effect size (*R*^2^ = 0.143, *R* = 0.378). Dotted line on the *x*-axis indicates target HbA1c of 7.0%. **b** Greater percent DNA methylation of CpG9 was also significantly associated with higher LDL (*β* = 0.344, *p* = 0.037) with small to medium effect size (*R*^2^ = 0.040, *R* = 0.201). Dotted line on the *x*-axis indicates target LDL of 100 mg/dL. **c** Greater percent methylation of the *FKBP5* intron 2 CpG7-dinucleotide (CpG7) (coordinates chr6: 35,609,628) was significantly associated with higher BMI (*β* = 0.516, *p* = 0.001) with medium to large effect size (*R*^2^ = 0.227, *R* = 0.476). Dotted line on the *x*-axis indicates a BMI cut-off for obesity at ≥ 30 kg/m^2^. **d** Greater percent DNA methylation of CpG7 was also significantly associated with higher WC (*β* = 0.403, *p* = 0.006) with medium to large effect size (*R*^2^ = 0.123, *R* = 0.350). Dotted lines on the *x*-axis indicate cut-offs for WC in metabolic syndrome criteria of ≥ 94 cm in men or ≥ 80 cm in women

We analyzed by pyrosequencing nine CpG-dinucleotides in intron 2 of *FKBP5* that were implicated in metabolic disorders in previous studies and flanked an experimentally validated GRE [8, 11]. We observed a significant association between intronic *FKBP5* DNAm in subjects with T2DM and multiple cardiometabolic outcomes in two different CpG-dinucleotides in intron 2 (Fig. 1). Greater percent DNAm of the *FKBP5* intron 2 CpG9-dinucleotide (CpG9) (coordinates chr6: 35,609,542) was significantly associated with higher HbA1c (*β* = 0.535, *p* = 0.003, Fig. 1a) and LDL cholesterol (*β* = 0.344, *p* = 0.037, Fig. 1b). Greater percent DNAm of the *FKBP5* intron 2 CpG7-dinucleotide (CpG7) (coordinates chr6: 35,609,628) was significantly associated with higher BMI and WC (*β* = 0.516, *p* = 0.001, Fig. 1c, and *β* = 0.403, *p* = 0.006, Fig. 1d, respectively). The associations with HbA1c, BMI, and WC were all of medium to large effect sizes (Table 2). These findings remained stable regardless of using the complete case sample (*n* = 43) or all available cases despite missing data (Additional file 2: Table S1, Additional file 3: Figure S2). Results for non-significant associations are shown in Additional file 2: Table S2.

**Table 2:** *FKBP5* methylation associated with cardiometabolic risk in individuals with diabetes

Independent variables	Model including the covariates age, sex, and race		Effect size	
	*β*	*p*	*R*	Effect size
Percent methylation of CpG9				
HbA1c	0.535	0.003**	0.378	Medium-large
LDL	0.344	0.037*	0.201	Small to medium
Percent methylation of CpG7				
BMI	0.516	0.001**	0.476	Large
WC	0.403	0.006**	0.350	Medium-large

The analysis included 43 subjects. Percent methylation of CpG9 and CpG7 were the dependent variables in the model

*p < 0.05; **p < 0.01

In a secondary analysis, it was observed that having a history of more invasive cardiovascular procedures was associated with greater percent DNAm at CpG9 when adjusting for LDL cholesterol (*β* = 0.344, *p* = 0.033) (Table 3). Greater percent DNAm at CpG7 was inversely associated with more days a week completing more than 30 min of physical activity even when controlling for WC (*β* = − 0.390, *p* = 0.011) (Table 3).

**Table 3:** *FKBP5* methylation associated with cardiovascular procedures and days of physical activity in individuals with diabetes

Dependent variable	Independent variable	Model including the covariates age, sex, and race	Model including additional covariate^a^
Number of cardiovascular procedures	Percent methylation of CpG9	*β* = 0.247, *p* = 0.112	LDL; *β* = 0.344, *p* = 0.033*
Percent methylation of CpG7	Number of days of physical activity	*β* = −0.492, *p* = 0.001**	WC; *β* = − 0.390, *p* = 0.011*

^a^This model includes covariates of age, sex, and race in addition to the covariate listed below

*p < 0.05; **p < 0.01

## Discussion

In our study of individuals with T2DM, we observed that one pyrosequenced CpG-dinucleotide in intron 2 of the *FKBP5* gene (CpG9) was significantly associated with higher HbA1c and LDL, and another CpG-dinucleotide (CpG7) site with BMI and WC, though results should be interpreted in the context of a small sample size of 43 subjects. In the study population, mean HbA1c was above 7% (8.3%), mean BMI was categorized as obese (> 30 kg/m^2^), there was a presence of hyperlipidemia (LDL cholesterol greater than 100 mg/dL) in 30% of subjects, and a majority of subjects (81.4%) were above target WC and thus met criteria for metabolic syndrome. This suggests that the associations between DNAm of *FKBP5* and the outcome measures for cardiovascular and metabolic dysfunction observed in our study population including HbA1c, LDL cholesterol, BMI, and WC, are clinically meaningful as risk for disease. The relationship between *FKBP5* methylation, a surrogate marker for presumed chronic cortisol exposure, glycemic control in T2DM, and cardiovascular disease (CVD) risk, is notably indicated by the medium to large effect size of the association between intron 2% DNAm with HbA1c and WC, and the medium effect size of the relationship with LDL cholesterol. Our secondary analysis further suggests that the association of *FKBP5* with CVD as methylation of *FKBP5* is also associated with cardiovascular interventions including catheterization, percutaneous intervention (PCI), and coronary artery bypass grafting (CABG), and inversely associated with exercise.

**Fig. 2:**
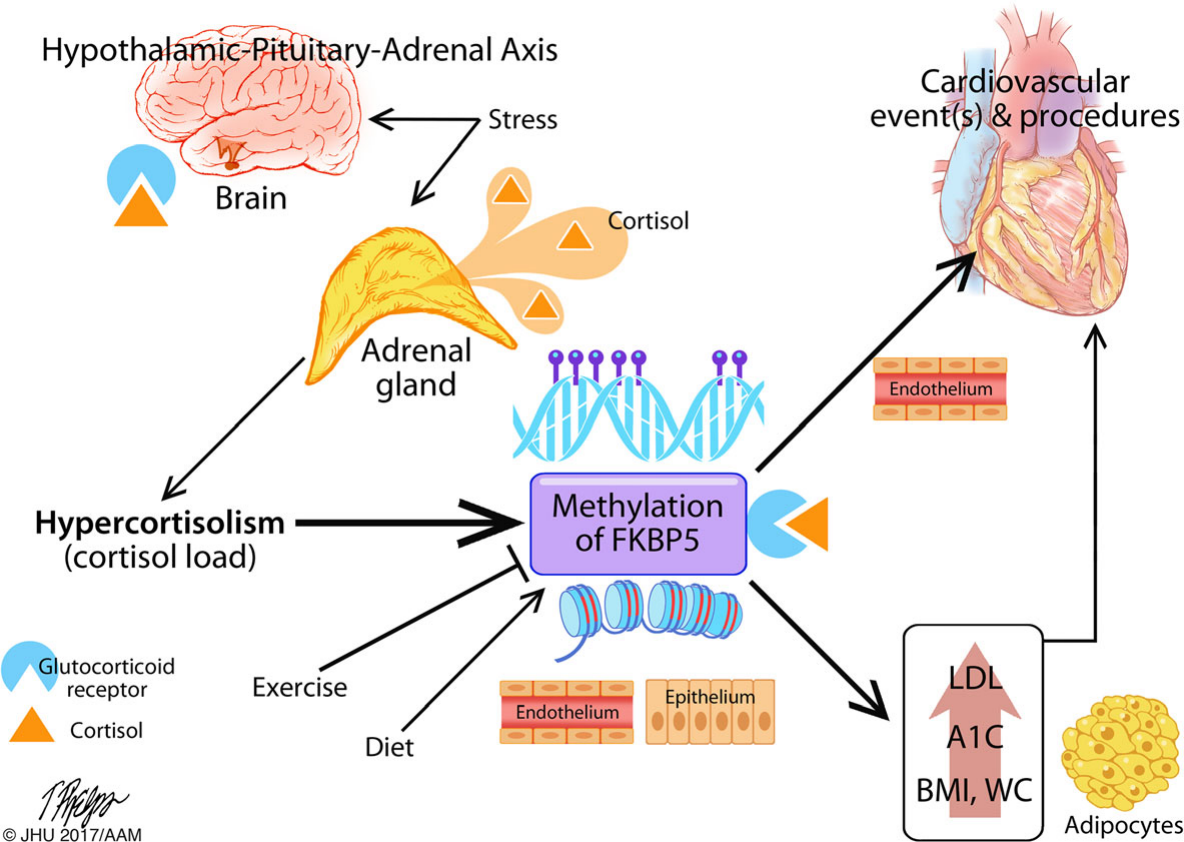
The hypothesized relationship of *FKBP5* DNA methylation and cardiometabolic risk. Our findings demonstrate evidence that *FKBP5*, and therefore likely the hypothalamic-pituitary-adrenal axis, play a role in metabolic and cardiovascular disease risk and outcome. *FKBP5*, and the glucocorticoid receptor, is expressed in the brain, intestinal endothelial, and epithelial tissues. *FKBP5* expression in the brain (hypothalamus and hippocampus) as well as these other tissues has been associated previously with the influences of stress and cortisol load, exercise, and diet. Prior demonstration has also shown the function of *FKBP5* to play a role in adipogenesis and in endothelial changes in myocardial infarction. Taken together, these associations are hypothesized to link to risk and outcomes in cardiometabolic disease through methylation of *FKBP5*, and therefore, potentially expression or sensitivity of the glucocorticoid receptor. The methylation of *FKBP5* is associated with increased clinical risk factors for disease including hyperlipidemia (elevated LDL), chronic hyperglycemia (elevated HbA1c), and obesity (high BMI and WC) in individuals with diabetes. Further, methylation of *FKBP5* may be associated with cardiovascular procedures and inversely associated with exercise independent of LDL and obesity. Though many clinical trials have established the classical model of pathophysiologic associations with diabetes and hyperglycemia, hyperlipidemia, obesity, and cardiometabolic disease, the findings illustrated here demonstrate that there may be a role for the hypothalamic-pituitary-adrenal axis and glucocorticoid pathway dysfunction in the underlying pathophysiology of cardiometabolic disease risk and outcomes

There is a higher incidence of CVD in individuals with diabetes due to both macrovascular pathology and associated comorbid metabolic disorders, including hyperlipidemia [20, 21]. However, current treatments continue to target individual disease processes. Though our findings are correlative and cannot be used to determine causality, they support our hypothesis of a potential unifying pathophysiology of underlying hypercortisolism related to activation of glucocorticoid receptor signaling (Fig. 2), which may implicate the glucocorticoid pathway as a new potential target for preventive and therapeutic interventions.

Our study corroborates current literature demonstrating the significance of *FKBP5* in associations with metabolic dysfunction and dysglycemia. For example, a polymorphism, rs1360780, has been associated with insulin resistance in children and adolescents with obesity and also with lower weight loss up to 26 months after bariatric surgery in adults [10, 22]. Studies have also demonstrated that *FKBP5* protein expression is responsive to certain environmental stimuli. For example, animal studies have shown that higher levels of *FKBP5* expression in the hypothalamus are related to increases in body weight and, further, hypothalamic, hippocampal, and intestinal colon epithelial expression of *FKBP5* may be responsive to dietary intake and environmental stress [23–25]. In humans, adipose tissue *FKBP5* expression and insulin resistance increase with dexamethasone exposure [9].

The observations in our secondary analysis that higher *FKBP5* methylation is inversely associated with current exercise activity and positively associated with a history of cardiovascular procedures are also supported by the literature. It has been demonstrated that *FKBP5* may play a role in vascular endothelial function in exercise in obese individuals as well as on endothelial platelet aggregation in individuals with acute myocardial infarction [26–28]. This dysfunction may be the underpinnings of the associations between cortisol and coronary artery calcifications, mortality after cardiac surgery (CABG), and all-cause CVD mortality [29–31]. Similarly, elevated hair cortisol levels as a measure of chronic cortisol exposure predict myocardial infarction and heart failure severity [32, 33].

Epigenetics is the concept that the genome may be modifiable through environmental exposures. One method of epigenetic modification is methylation of CpG-dinucleotide sites of DNA. One study demonstrated that a decrease in methylation of an intron 2 site of *FKBP5* is present in Cushing’s syndrome [11]. Only one study to our knowledge, an animal study, exists assessing methylation and expression of *FKBP5* as a result of chronic steroid exposure, which showed persistent loss of methylation correlating with increased steroid exposure [4]. These studies suggest hypomethylation in association with chronic glucocorticoid exposure and our study identified hypermethylation associated with risk factors for CVD in T2DM. However, we note that two of the *FKBP5* intronic CpGs analyzed by Klengel et al. also showed increase in DNAm associated with the trauma exposure [8]. While gain of methylation events are usually associated with gene silencing, there are notable examples, such as at imprinted loci, where increase in DNAm inhibit binding of insulator proteins such as CTCF that govern complex enhancer-promoter interactions and gene activation [34]. If lower *FKBP5* expression is the outcome of hypermethylation in this group, it could indicate that these subjects are more responsive at the tissue level to cortisol since lower *FKBP5* protein expression allows more cortisol signaling. The increased responsiveness in adipose and other tissues could be the reason for the correlation [9, 24, 25, 28]. In this scenario, methylation is not a marker of cortisol exposure but of cortisol sensitivity. Additional in vitro experiments are needed to identify methylation-dependent binding of disease-specific factors whose presence in intron 2 may increase expression of *FKBP5* and its relationship with chronicity of cortisol exposure. Though further studies are needed to elucidate these gene loci-specific environment interactions, taken together with the findings in the current literature, our study may suggest that chronic exposure to glucocorticoids in obesity and T2DM may be in part responsible for associated cardiovascular and metabolic dysfunction through *FKBP5* expression.

Our study has many strengths. First, our sample includes a clinically generalizable sample of patients with T2DM and clinical outcome measures relevant to the assessment of disease in this population, especially HbA1c as a marker of glucose control, LDL cholesterol as a marker of increased risk of CVD, and BMI and WC as markers of a diagnosis of metabolic syndrome. Second, our study is one of the first studies to explore the epigenetics of *FKBP5* in humans with T2DM, in relation to metabolic and CVD risk and clinical characteristics including exercise and cardiac procedures. Finally, there is strength in the specificity of selecting a region of interest within the *FKBP5* gene, intron 2. This offers a specific target for future studies and potentially future interventions.

Our study does have some potential limitations. First, this is a small sample size and only includes individuals with T2DM seen in a tertiary care practice without a control group, which limits generalizability. The small sample size also limits the ability to adjust for multiple confounders and permits risk of overfitting the data in analyses. Given the small sample size and that the *FKBP5* analysis was hypothesis generating as a secondary aim of the MinD study, it may have been underpowered to accurately assess the effect sizes listed in Table 2. Also, control subjects without T2DM were not included in the original design. Future studies should include larger samples with control subjects in order to more stringently establish, and then validate, methylation as a potential biomarker for chronic glucocorticoid exposure. Second, our study is correlative and does not offer insight into causative association between glucocorticoid receptor function and disease outcomes. Third, we do not have longitudinal cortisol measures to assess the association with percent methylation as a surrogate for chronic hyper- or hypocortisolism nor did we measure peripheral *FKBP5* protein expression and, therefore, cannot assess whether the increase in DNA methylation we observe is associated with *FKBP5* or cortisol levels. Therefore, we propose that future studies of greater sample size, including a matched control group, assess both chronic, longitudinal assessment of cortisol load, such as hair cortisol levels, and RNA expression of *FKBP5* protein. We also assessed peripheral blood samples for *FKBP5* methylation and not adipocytes, epithelial, or endothelial cells. Finally, in our statistical analyses, our associations were interpreted without corrections for multiple comparisons, as typical multiple corrections assume that the tests are independent and are too conservative for correlated hypotheses as we have tested here.

## Conclusion

In conclusion, we examined *FKBP5* methylation among individuals with T2DM and found that *FKBP5* DNAm was associated with higher levels of HbA1c, LDL cholesterol, BMI, and WC, which are all risk factors for CVD. We also found that increased percent *FKBP5* methylation was associated with greater number of cardiac procedures, supporting our initial finding of the relationship between *FKBP5* and adverse cardiovascular risk factors. Thus, *FKBP5* DNAm may be a marker of higher cardiovascular risk in T2DM, possibly secondary to higher exposure to cortisol. There is a critical need for further characterization of the longitudinal association of *FKBP5* with CVD in T2DM as a first step in understanding potential causation, and future directions for preventive and treatment interventions targeting the HPA axis.

## Additional files

Additional file 1: **Figure S1.** Consort diagram. (PPTX 18 kb)

Additional file 2: **Table S1.** *FKBP5* methylation associated with cardiometabolic risk does not differ by missing data case exclusion. **Table S2.** *FKBP5* percent methylation at various CpG-dinucleotide sites in intron 2 is not associated with cardiometabolic risk. (DOCX 20 kb)

Additional file 3: **Figure S2.** *FKBP5* methylation associated with cardiometabolic risk in individuals with diabetes including all available data. (PPTX 28 kb)

## Abbreviations

BMI: Body mass index (kg/m^2^)
BP: Blood pressure
CABG: Coronary artery bypass graft
Chr: Chromosome
CpG: Cytosine guanine dinucleotide
CVD: Cardiovascular disease
DNA: Deoxyribonucleic acid
DNAm: DNA methylation
*FKBP5*: FK506-binding protein 51 kDa gene
GRE: Glucocorticoid response element
HbA1c: Glycosylated hemoglobin (%)
HPA: Hypothalamic-pituitary-adrenal
HPLC: High-performance liquid chromatography
LDL: Low-density lipoprotein cholesterol (mg/dL)
MinD: Minor depressive disorder
PCR: Polymerase chain reaction
T2DM: Type 2 diabetes mellitus
TG: Triglycerides
WC: Waist circumference (cm)
