# Supplementary material for: Type 2 diabetes and cardiometabolic risk may be associated with increase in DNA methylation of FKBP5
Source: Clin Epigenetics. 2018 Jun 19;10:82. doi: 10.1186/s13148-018-0513-0 (PMC6010037; doi:10.1186/s13148-018-0513-0)
Supplement: Supplementary file 1 — Figure S1. Consort diagram. (PPTX 18 kb) [file 13148_2018_513_MOESM1_ESM.pptx]

## Slide 1
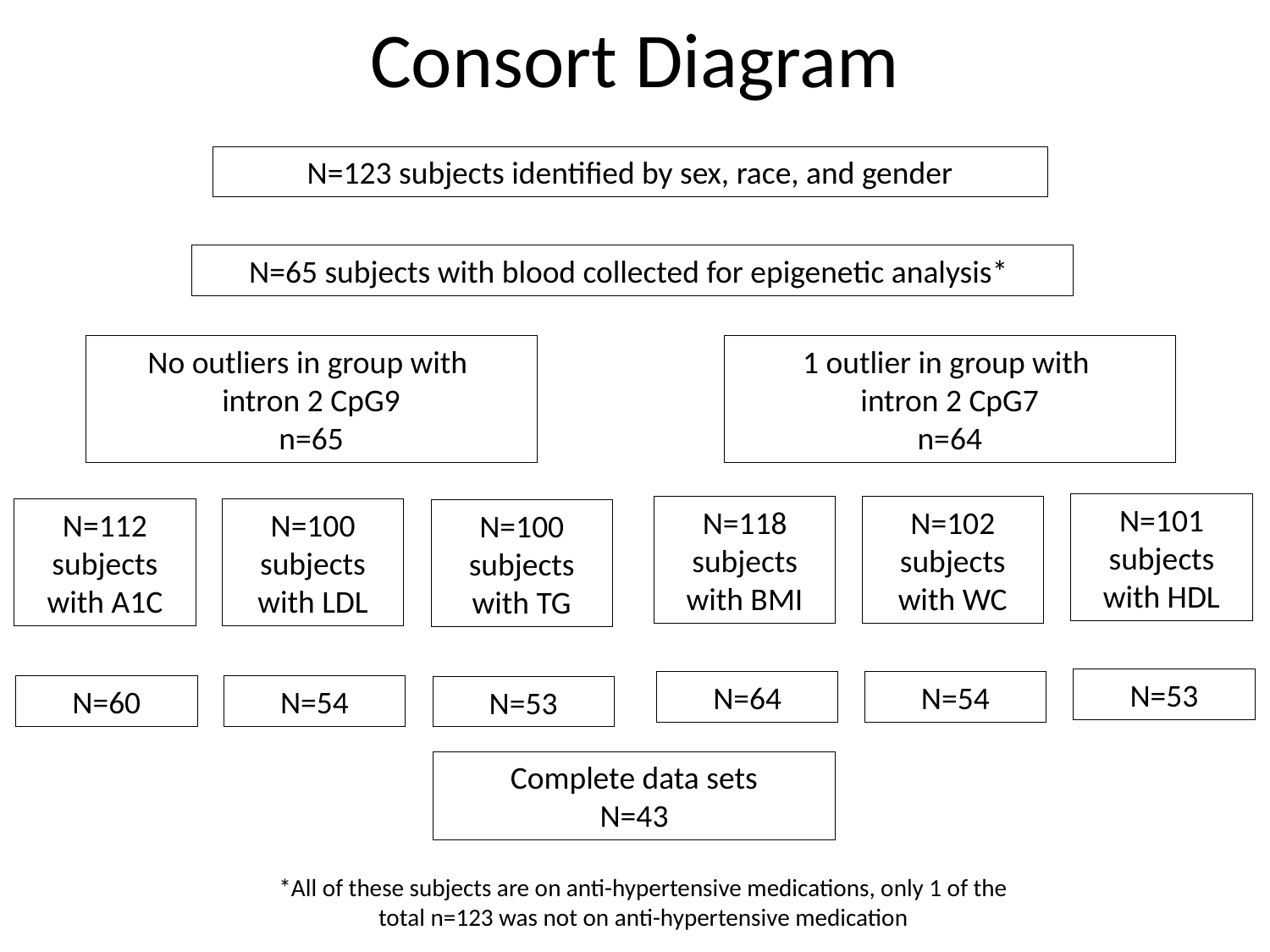

# Consort Diagram
N=123 subjects identified by sex, race, and gender
N=65 subjects with blood collected for epigenetic analysis*
No outliers in group with
intron 2 CpG9
n=65
1 outlier in group with
intron 2 CpG7
n=64
N=101 subjects with HDL
N=118 subjects with BMI
N=102 subjects with WC
N=112 subjects with A1C
N=100 subjects with LDL
N=100 subjects with TG
N=53
N=64
N=54
N=60
N=54
N=53
Complete data sets
N=43
*All of these subjects are on anti-hypertensive medications, only 1 of the total n=123 was not on anti-hypertensive medication
