# Supplementary material for: Type 2 diabetes and cardiometabolic risk may be associated with increase in DNA methylation of FKBP5
Source: Clin Epigenetics. 2018 Jun 19;10:82. doi: 10.1186/s13148-018-0513-0 (PMC6010037; doi:10.1186/s13148-018-0513-0)
Supplement: Supplementary file 2 — Table S1. FKBP5 methylation associated with cardiometabolic risk does not differ by missing data case exclusion. Table S2. FKBP5 percent methylation at various CpG-dinucleotide sites in intron 2 is not associated with cardiometabolic risk. (DOCX 20 kb) [file 13148_2018_513_MOESM2_ESM.docx]

Additional file 2: Table S1. *FKBP5* methylation associated with cardiometabolic risk does not differ by missing data case exclusion

| Independent variables | Model including the covariates age, sex, and race | | Effect size | |
| --- | --- | --- | --- | --- |
|  | β | p | R | Effect size |
| Methylation of CpG9 | | | | |
| HbA1c (n=60) | 0.350 | 0.014* | 0.312 | Medium to large |
| LDL (n=54) | 0.350 | 0.014* | 0.184 | Small to medium |
| Methylation of CpG7 | | | | |
| BMI (n=64) | 0.352 | 0.010* | 0.307 | Medium |
| WC (n=44) | 0.374 | 0.007** | 0.360 | Medium to large |

*p<0.05, **p<0.01

Additional file 2: Table S2. *FKBP5* percent methylation at various CpG-dinucleotide sites in intron 2 not associated with cardiometabolic risk

|  | CpG1 | CpG2 | CpG3 | CpG4 | CpG5 | CpG6 | CpG8 |
| --- | --- | --- | --- | --- | --- | --- | --- |
| HbA1c | β=0.128,  p=0.558 | β=-0.038, p=0.860 | β=-0.309, p=0.143 | β=-0.402, p=0.053 | β=0.133, p=0.488 | β=0.129, p=0.509 | β=0.280, p=0.161 |
| LDL | β=-0.096,  p=0.620 | β=0.217, p=0.253 | β=-0.125, p=0.510 | β=-0.215, p=0.258 | β=0.127, p=0.459 | β=0.265, p=0.125 | β=-0.173, p=0.340 |
| WC* | β=0.042,  p=0.822 | β=0.028, p=0.878 | β=-0.258, p=0.149 | β=-0.011, p=0.951 | β=-0.102, p=0.526 | β=0.031, p=0.848 | β=-0.037, p=0.824 |

N=43, model includes covariates of age, sex, and race

*Given the significant correlation between BMI and WC (R=0.73, p<0.0001), and effort to reduce multiple comparisons the original analysis included WC only for these variables
