# Supplementary material for: Type 2 diabetes and cardiometabolic risk may be associated with increase in DNA methylation of FKBP5
Source: Clin Epigenetics. 2018 Jun 19;10:82. doi: 10.1186/s13148-018-0513-0 (PMC6010037; doi:10.1186/s13148-018-0513-0)
Supplement: Supplementary file 3 — Figure S2. FKBP5 methylation associated with cardiometabolic risk in individuals with diabetes including all available data. (PPTX 28 kb) [file 13148_2018_513_MOESM3_ESM.pptx]

## Slide 1
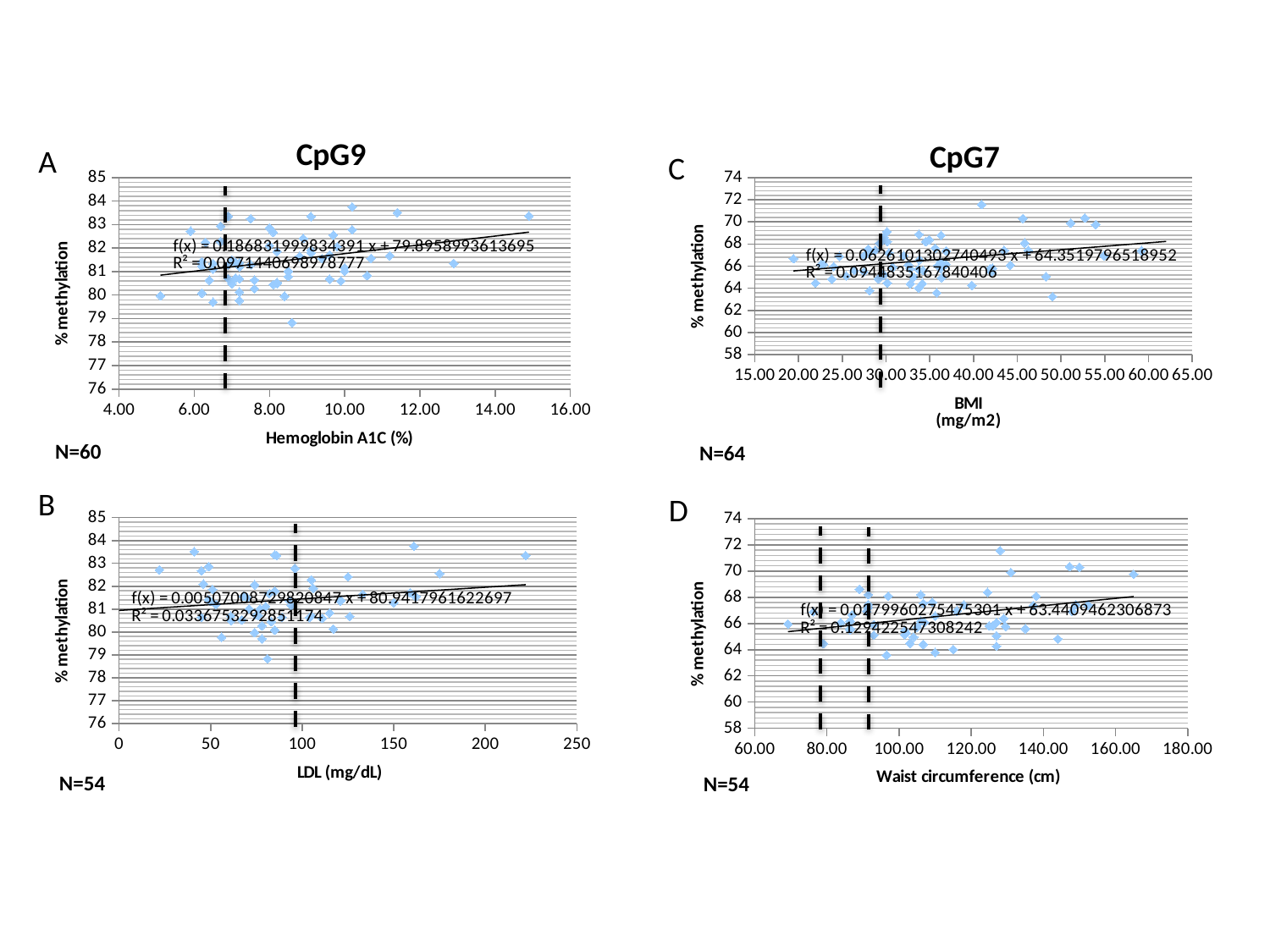

CpG9
CpG7
A
C
### Chart
| Category | hFKint2Region4Pyro2CpG5 |
|---|---|
### Chart
| Category | hFKint2Region4Pyro1CpG3 |
|---|---|N=60
N=64
B
D
### Chart
| Category | hFKint2Region4Pyro2CpG5 |
|---|---|
### Chart
| Category | hFKint2Region4Pyro1CpG3 |
|---|---|N=54
N=54
